# Upfront Management of Blastoid Variant Mantle Cell Lymphoma: Making the Case for 2nd/3rd‐Generation Bruton Tyrosine Kinase Inhibitor‐Based Therapies

**DOI:** 10.1002/cam4.70310

**Published:** 2024-10-12

**Authors:** Benjamin J. Lee, Jenny Liu, Shawn P. Griffin, Jean Doh, Stefan O. Ciurea, Piyanuch Kongtim, Elizabeth A. Brem

**Affiliations:** ^1^ Department of Pharmacy University of California Irvine Health Orange California USA; ^2^ Department of Clinical Pharmacy Practice School of Pharmacy & Pharmaceutical Sciences, University of California Irvine California USA; ^3^ Department of Medicine, Division of Hematology Oncology, Chao Family Comprehensive Cancer Center University of California Irvine Health Orange California USA

**Keywords:** blastoid variant, Bruton tyrosine kinase inhibitor, mantle cell lymphoma

## Abstract

**Introduction:**

Blastoid variant‐mantle cell lymphoma (BV‐MCL) represents an aggressive subset of patients with no established standard of care treatment approach.

**Methods:**

We performed a retrospective analysis of the clinical characteristics and outcomes of 17 de novo BV‐MCL patients treated at our institution between May 2009 and September 2023. In addition, we reviewed the literature supporting 2nd/3rd generation Bruton's Tyrosine‐kinase (BTKi)‐based therapies for upfront management.

**Results:**

The complete response rate to frontline and salvage therapy was 66.7% and 25%, respectively. Two‐year overall survival (OS) was low at 62.5% (95% CI, 34.7%–81.1%). Variables associated with higher OS included receipt of consolidative HSCT (*p* = 0.017), TP53‐wild type (*p* = 0.031), intermediate‐ versus high‐risk Mantle Cell Lymphoma Prognostic Index score (*p* = 0.026), and achieving complete response with induction therapy (*p* = 0.027).

**Discussion:**

Treatment outcomes with chemo‐immunotherapy in BV‐MCL patients are poor, especially among TP53‐mutated patients. Recent findings describing positive outcomes with novel BTKi‐based therapies are encouraging and should be considered in this high‐risk population.

## 1 Introduction

Mantle cell lymphoma (MCL) is a rare subtype of non‐Hodgkin's lymphoma that is uniquely characterized by t(11; 14)(q13; q32) which leads to overexpression of cyclin D1. Although its clinical course is widely heterogenous, the 10%–20% of patients with pleomorphic or blastoid variant (BV)‐MCL have suboptimal survival outcomes with no clear guidance on appropriate upfront therapy [1, 2]. Recent advancements in targeted small molecule therapies, such as Bruton tyrosine kinase inhibitors (BTKi) and the BCL‐2 inhibitor, venetoclax, have changed the treatment landscape for relapsed/refractory (R/R) MCL resulting in significantly improved survival outcomes.

Early incorporation of these novel targeted therapies for the management of BV‐MCL may improve long‐term outcomes for this highly aggressive subset of patients. Response to upfront and subsequent lines of therapy for BV‐MCL however, is not well characterized in the literature so optimal sequencing and utilization is currently unknown. The objective of this study is to describe the treatment course and outcomes of de novo BV‐MCL patients treated at our institution. In addition, we evaluate published literature describing the BTKi therapies, acalabrutinib and zanubrutinib, for upfront management.

## 2 Methods

Adult patients (18 years of age or older) with untreated BV‐MCL between May 2009 and September 2023 were included in this institution review board‐approved study. All patients underwent baseline ^18^F‐fluorodeoxyglucose (FDG) positron emission tomography with computed tomography (PET‐CT) imaging and bone marrow biopsy testing for initial staging. The primary outcome was complete response (CR) by PET‐CT assessed in accordance with the Lugano criteria [3]. Secondary outcomes included progression‐free (PFS) and overall survival (OS). PFS was defined as the time from treatment initiation to disease progression or death, whichever occurred first. Time‐to‐event analyses were summarized via the Kaplan–Meier method, and treatment groups were compared using a two‐sided log‐rank test. Assessment of consolidative autologous hematopoietic stem cell transplantation (aHSCT; within 6 months of treatment initiation) was performed utilizing landmark analysis restricted to patients without events before 6 months.

## 3 Results

A total of 17 patients were identified. The median age was 74 years (range, 52–99) and 76.5% were male. Sixteen patients (94.1%) had a high proliferative index (≥ 40%) with a median Ki‐67 of 75% (range, 30%–100%) and the majority had stage IV disease (76.5%). All patients had extranodal involvement of their MCL with bone marrow (52.9%; *n* = 9/17) and thoracic (41.2%; *n* = 7/17) sites being the most frequent. The median Mantle Cell Lymphoma Prognostic Index (MIPI) score was 8 (range, 5.9–10.8) and 76.5% of patients were categorized as high‐risk while the remaining 23.5% were intermediate‐risk (Table 1). Cytogenetic testing was available in 14 patients, of whom 10 (71.4%) had a TP53 mutation. Fifteen patients (88.2%) received an upfront rituximab‐based chemotherapy regimen: 46.7% (*n* = 7) including bendamustine‐rituximab (BR), 13.3% (*n* = 2) BR followed by BR and cytarabine, 13.3% (*n* = 2) R‐CHOP, 13.3% (*n* = 2) R‐HyperCVAD, 6.7% (*n* = 1) R‐EPOCH, and 6.7% (*n* = 1) received the Nordic regimen (R‐maxi‐CHOP alternating with high‐dose cytarabine). One patient received venetoclax monotherapy while another acalabrutinib with obinutuzumab.

**TABLE 1:** Baseline characteristics.

Variables^a^	All patients (*n* = 17)	TP53‐mutated (*n* = 10)	TP53 wild type (*n* = 4)	Unknown TP53 status (*n* = 3)
Male, no. (%)	13 (76.5)	8 (80)	3 (75)	2 (66.7)
Age (year)	74 (52–99)	77 (52–86)	68 (53–74)	72 (57–99)
ECOG PS, no. (%)				
0–1	10 (58.8)	6 (60)	4 (100)	0 (0)
2–4	7 (41.2)	4 (40)	0 (0)	3 (100)
Stage, no. (%)				
Stage I–II	2 (11.8)	1 (10)	0 (0)	1 (33.3)
Stage III–IV	15 (88.2)	9 (90)	4 (100)	2 (66.7)
Number of extranodal sites	2 (2–3)	3 (2–3)	2 (2–3)	2 (2–3)
Extranodal sites ≥ 3, no. (%)	7 (41.2)	5 (50)	1 (25)	1 (33.3)
BM involvement, no. (%)	10 (58.8)	6 (60)	3 (75)	1 (33.3)
CNS involvement, no. (%)	0 (0)	0 (0)	0 (0)	0 (0)
Ki‐67 (%)	75 (30–100)	85 (40–100)	55 (30–85)	70 (50–85)
MIPI score, no. (%)				
Low‐risk MIPI	0 (0)	0 (0)	0 (0)	0 (0)
Intermediate‐risk MIPI	4 (23.5)	2 (20)	2 (50)	0 (0)
High‐risk MIPI	13 (76.5)	8 (80)	2 (50)	3 (100)

Abbreviations: BM, bone marrow; CNS, central nervous system; ECOG, Eastern Cooperative Oncology Group; MIPI, mantle cell lymphoma international prognostic index; PS, performance status.

^a^ All variables reported as median (range) unless otherwise stated.

**FIGURE 1:**
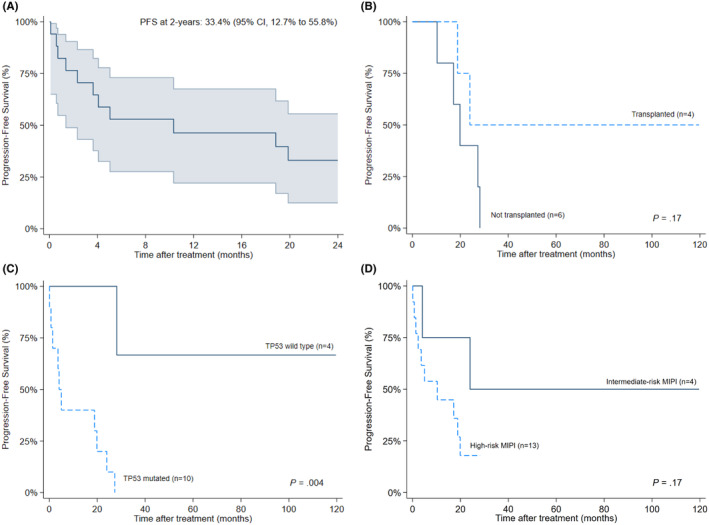
Kaplan–Meier plots for progression‐free survival (PFS) among (A) all included patients at 2 years, and between patients (B) who received or did not receive consolidative HSCT, (C) with or without known TP53‐mutation, and (D) with high‐ versus intermediate‐risk Mantle Cell Lymphoma International Prognostic Index scores.

The CR rate with frontline therapy was 64.7% (*n* = 11/17), with 50% of TP53‐mutated patients achieving response (including one patient who received acalabrutinib and obinutuzumab). There was no significant difference in CR rates between patients with high versus intermediate MIPI at baseline (61.5% [*n* = 8/13] vs. 75% [*n* = 3/4]; *p* > 0.99). Four patients proceeded to aHSCT. The remaining seven transitioned to maintenance therapy with rituximab (*n* = 4), BR (*n* = 1), or acalabrutinib (*n* = 2). At a median follow‐up of 24.7 months (range, 0.1–119.6), median PFS was 10.3 months and two‐years PFS was 33.4% (95% CI, 12.7%–55.8%) (Figure 1A). Variables associated with worse PFS included the presence of a TP53‐mutation (*p* = 0.004) but not consolidative HSCT (*p* = 0.17) or higher risk MIPI (*p* = 0.17) (Figure 1B–1D).

**FIGURE 2:**
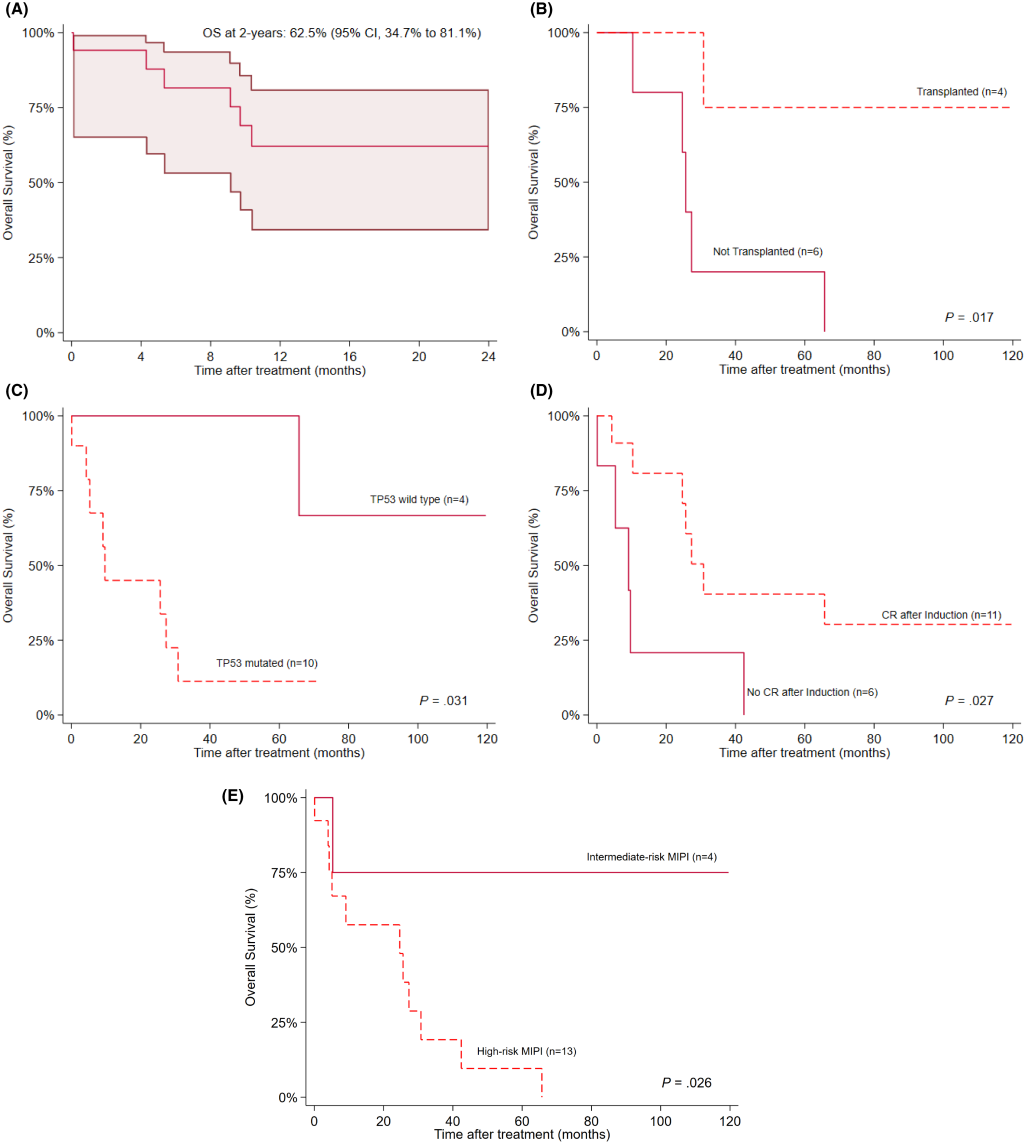
Kaplan–Meier plots for overall survival (OS) among (A) all included patients at 2 years and between patients (B) who received or did not receive consolidative HSCT, (C) with or without known TP53‐mutation, (D) achieved or did not achieve CR with induction therapy, and (E) with high‐ versus intermediate‐risk Mantle Cell Lymphoma International Prognostic Index scores.

Ten patients received second‐line therapy, of whom two were not evaluable due to early disease‐related mortality. Six had refractory disease, leading to a CR rate of 25% (*n* = 2/8). One patient subsequently relapsed at 5.3 months and was initiated on ibrutinib monotherapy. This patient relapsed two additional times, ultimately passing from disease progression 3 months after receipt of CD19 chimeric antigen receptor T‐cell therapy. The second patient achieved a CR on acalabrutinib monotherapy and was subsequently bridged to an allogeneic HSCT. This patient remains disease free 3 years later on acalabrutinib maintenance. Of the remaining patients totaling 6 courses of third or later lines of therapy received, two (33.3%) resulted in short‐term CR (< 6 months). Radiotherapy was utilized in three instances for salvage treatment of which all three failed to achieve a response. Two‐year OS for the whole cohort was 62.5% (95% CI, 34.7%–81.1%) (Figure 2A). OS was significantly higher in patients who received consolidative HSCT (*p* = 0.017), did not have a TP53‐mutation (*p* = 0.031), had intermediate‐ versus high‐risk MIPI (*p* = 0.026), and achieved a CR with induction therapy (*p* = 0.027) (Figure 2B–E).

## 4 Discussion

BV‐MCL represents an aggressive subtype with poor long‐term survival that is highly refractory to chemo‐immunotherapy, in part due to the prevalence of the TP53 mutation [1, 2]. Consistent with features of MCL, the median age of our cohort was 74 years. Of those who responded, only 36.4% proceeded to HSCT. With a 2‐year PFS and OS of 33.4% and 62.5%, respectively, it is evident that a more efficacious therapeutic approach is needed. While preferred treatment approaches remain undefined, the introduction of BTKi's revolutionized the treatment paradigm for R/R MCL.

The SHINE study demonstrated significantly prolonged PFS in patients who received ibrutinib plus BR versus BR alone (HR = 0.75; 95% CI, 0.59–0.96; *p* = 0.01), although this benefit was not evident among those with a blastoid or pleomorphic histology (HR = 0.66; 95% CI, 0.32–1.35) or TP53 mutation (HR = 0.95; 95% CI, 0.50–1.80) [4]. Despite a significant improvement in PFS over SOC, OS at 7 years was similar, and a higher rate of grade ≥ 3 toxicity was reported with ibrutinib (81.5% vs. 77.3%). The SHINE study, however, does not address the question of whether a BTKi alone results in improved outcomes. An ongoing study of zanubrutinib and rituximab vs. BR in patients with newly diagnosed MCL may shed light on the role of BTKi for upfront treatment (NCT04002297) [5].

### 4.1 BTKi‐Based Therapies for R/R BV‐MCL

Subsequent approvals of 2nd‐generation BTKi therapies with decreased cardiac morbidities are promising upfront therapies for BV‐MCL. The ACE‐LY‐004 trial was an open‐label, multicenter, study of oral acalabrutinib monotherapy for R/R MCL [6]. With a median follow‐up of 38.1 months, an 89% overall response rate (ORR) with a 40% CR rate was achieved for all included patients. Duration of response (DOR) was 28.6 months with a PFS of 22 months. Significantly, in a post hoc analysis of the ACE‐LY‐004 study evaluating patients with blastoid or pleomorphic disease, investigators reported an ORR of 88% (95% CI, 61.7%–98.4%) in patients with 1 prior line of therapy with a DOR of 20.3 months (95% CI, 3.4–46.9) [7]. Zanubrutinib was evaluated in a multicenter, single‐arm study of 86 patients with R/R MCL [8]. It too demonstrated impressive findings with an ORR of 83.7% and a CR rate of 77.9%. In a subgroup analysis of BV histology, investigators reported an ORR of 66.7% with similar DOR (30.6 vs. 30.2 months) and PFS (25 vs. 27.8 months) compared to those with a classic MCL histology. The recent approval of pirtobrutinib, a non‐covalent BTKi, expands the potential armamentarium for BV‐MCL [9]. With an ORR of 75% (95% CI, 34.9–96.8) among included BV‐MCL patients (*n* = 9) in the BRUIN trial, future studies are warranted to evaluate this potential drug therapy.

The utility and place in therapy for combination BTKi and venetoclax with or without a CD20‐monoclonal antibody also needs to be considered for this population. The recently presented SYMPATICO trial demonstrated improved PFS with ibrutinib and venetoclax over ibrutinib alone in R/R MCL patients, including the BV‐MCL subgroup [10]. In another recently presented abstract, the combination of acalabrutinib, venetoclax, and rituximab produced a CR rate of 67% in three R/R BV‐MCL patients [11]. Early evidence supporting dual BTKi and venetoclax therapy is encouraging, although more mature data is required.

### 4.2 BTKi‐Based Therapies for Upfront MCL

While the ACE‐LY‐004, BGB‐3111‐206, and BRUIN trials demonstrated efficacy of acalabrutinib, zanubrutinib, and pirtobrutinib, respectively, in R/R BV‐MCL, one recent study suggests that upfront BTKi‐based therapies may be the key in improving outcomes in this difficult to treat population. In a large longitudinal database analysis of 3771 MCL patients, OS with a BTKi in the first, second, and third or later line setting was 35 months (95% CI, 27–50), 24 months (95% CI, 22–30), and 18 months (95% CI, 14–21), respectively [12]. As OS progressively shortens with later‐line BTKi use, it is evident that upfront BTKi treatment should be considered. Moreover, the utilization of a BTKi mitigated the negative impact of del 17p/TP53 mutation in this study, further supporting upfront use in BV‐MCL given the high predominance of TP53 mutation in these patients. For BV‐MCL patients fit for consolidative HSCT, achieving a CR with induction therapy and promptly proceeding to transplantation is imperative. In one of the largest studies evaluating BV‐MCL patients in the rituximab era, receipt of consolidative HSCT (HR = 0.52; 95% CI, 0.31–0.80; *p* < 0.01) and achieving CR with induction therapy (HR = 0.29; 95% CI, 0.17–0.51; *p* < 0.01) were associated with improved PFS. Attainment of CR with induction therapy was also significant in improving OS [13].

To contextualize the findings of the above‐reported studies, several ongoing investigational trials have recently described compelling outcomes. In a single‐arm, phase 2 trial of acalabrutinib and rituximab for older, untreated MCL patients, an impressive 94% ORR (90% CR) was achieved among 49 evaluable patients, including 3 with blastoid and 1 with pleomorphic disease [14]. The median PFS and OS were not yet reached, although 2‐year PFS and OS rates were 92% and 96%, respectively. From another cohort of 25 TP53‐mutated, de novo MCL patients (5 with BV‐MCL), Kumar, et al. described a 95% ORR (88% CR) with the BTKi combination of zanubrutinib, obinutuzumab, and venetoclax (BOVen) (NCT03824483) [15]. A phase 2 multicenter study evaluating acalabrutinib, venetoclax, and rituximab is also underway (NCT 05951959) [16]. Two ongoing studies investigating acalabrutinib/zanubrutinib and rituximab followed by consolidative HSCT may soon also shed light on the value of this approach to achieve long‐term survival outcomes in this challenging population (ACTRN12619000990123 and NCT05504603) [17, 18].

CD19‐directed chimeric antigen receptor T‐cell therapy (CAR‐T) is another novel immunotherapy approach that has recently demonstrated high response rates among R/R BV‐MCL patients in the pivotal ZUMA‐2 trial (92.9%; *n* = 13/14) [19]. As the long‐term outcomes of CAR‐T treatment in this population are currently unknown, more mature data is required to determine its optimal place in therapy for BV‐MCL.

## 5 Conclusion

In summary, our retrospective analysis demonstrates the need for improved first‐line therapy for patients with BV‐MCL. The evidence is compelling for the usage of 2nd/3rd‐generation BTKi‐based therapies with their touted efficacy in R/R BV‐MCL and improved toxicity profile compared to the first‐generation BTKi, ibrutinib. As upfront response to chemo‐immunotherapy is poor, especially among TP53‐mutated patients, and success with subsequent lines of therapy is low, 2nd/3rd‐generation BTKi‐based therapies and a CD20‐monoclonal antibody with or without venetoclax should strongly be considered in the front‐line setting. Future studies comparing outcomes in BV‐MCL patients receiving a 2nd/3rd‐generation BTKi regimen versus SOC chemo‐immunotherapy are needed to validate this approach.

## Author Contributions

**Benjamin J. Lee:** conceptualization (lead), data curation (lead), formal analysis (lead), investigation (equal), methodology (equal), writing – original draft (lead), writing – review and editing (equal). **Jenny Liu:** conceptualization (equal), formal analysis (equal), investigation (equal), methodology (equal), writing – original draft (equal), writing – review and editing (equal). **Shawn P. Griffin:** conceptualization (equal), formal analysis (equal), investigation (equal), methodology (equal), writing – review and editing (equal). **Jean Doh:** formal analysis (equal), investigation (equal), methodology (equal), writing – review and editing (equal). **Stefan O. Ciurea:** formal analysis (equal), investigation (equal), methodology (equal), resources (equal), writing – review and editing (equal). **Piyanuch Kongtim:** formal analysis (equal), investigation (equal), methodology (equal), resources (equal), writing – review and editing (equal). **Elizabeth A. Brem:** formal analysis (equal), investigation (equal), methodology (equal), resources (equal), supervision (equal), writing – review and editing (equal).

## Ethics Statement

This research was approved by the University of California Irvine Institutional Review Board (IRB #3877) and a waiver of informed consent was granted.

## Consent

Informed consent was waived in accordance with University of California Irvine Office of Research policies as no patient‐identifiable images or data have been included in this manuscript.

## Conflicts of Interest

Elizabeth A. Brem has received consulting fees from Astra Zeneca and BeiGene. Elizabeth A. Brem has received honoraria from Astra Zeneca, BeiGene, and AbbVie. The remaining authors declare no conflicts of interest.

## Data Availability

The data that support the findings of this study are available from the corresponding author upon reasonable request.
